# A novel digitonin/graphene oxide/iron oxide nanocomposite: synthesis, physiochemical characterization and antioxidant activity

**DOI:** 10.1186/s11671-024-03960-7

**Published:** 2024-01-22

**Authors:** Bashar Aljawrneh, Khaled Shawakfeh, Borhan Aldeen Albiss, Abdelelah Alshanableh, Mahmoud A. Al-Qudah, Tariq T. Bataineh, Lona Shawakfeh

**Affiliations:** 1 Department of Physics, Al-Zaytoonah University of Jordan, P.O. Box 130, Amman, 11733 Jordan; 2 Department of Chemistry, Jordan University of Science and Technology, P.O. Box 3030, Irbid, 22110 Jordan; 3 Nanotechnology Institute, Jordan University of Science and Technology, P.O. Box 3030, Irbid, 22110 Jordan; 4 Department of Chemistry, Faculty of Science, Yarmouk University, Irbid, Jordan; 5 The Joint School of Nanoscience and Nanoengineering, University of North Carolina at Greensboro, Greensboro, NC 27401 USA

**Keywords:** Graphene oxide, Iron magnetite nanoparticles, Digitonin, Functionalization, Antioxidant activity

## Abstract

In this work, iron oxide (Fe_3_O_4_) magnetic nanoparticles (MNPs) and graphene oxide (GO) nanosheets were prepared via the co-precipitation technique and the Modified Hummer method. Fe_3_O_4_ MNPs and GO nanosheets were combined to prepare Fe_3_O_4_/GO nanocomposite and subsequently conjugated with Digitonin (DIG) in order to obtain a dual-targeted delivery system based on DIG/Fe_3_O_4_/GO nanocomposite. SEM images reveal the presence of Fe_3_O_4_ MNPs at a scale of 100 nm, exhibiting dispersion between the GO nanosheets. Aggregation of the DIG/Fe_3_O_4_/GO nanocomposite was observed at various size scales. The XRD structural analysis confirms the crystal structure of the prepared samples. The Fe_3_O_4_ MNPs demonstrated the main XRD-diffracted peaks. Also, GO nanosheets exhibit crystalline characteristics on the (001) and (002) planes. The predominant peaks observed in the DIG/GO/Fe_3_O_4_ nanocomposite are attributed to the crystal phases of Fe_3_O_4_ MNPs. The FT-IR vibrational modes observed in the GO/DIG/Fe_3_O_4_ nanocomposite indicate the presence of crosslinking between GO nanosheet layers and the Fe_3_O_4_ MNPs. The antioxidant activity of the prepared samples was measured and the DIG/GO/Fe_3_O_4_ nanocomposite demonstrated a significantly high antioxidant activity in both 2-diphenyl-1-picrylhydrazyl (DPPH^·^) and 2,2-azino-bis-3-ethylbenzthiazoline-6-sulfonic acid (ABTS^·+^) tests.

## Introduction

Antioxidant activity refers to the ability to restrict or prevent nutritional oxidation through oxidative chain reactions [1, 2]. Significant research in the field of nanobiotechnology has recently been conducted, with a particular focus on the investigation of antioxidant activities [3–5]. Antioxidants that mitigate oxidative stress are in increasing demand as a form of preventative therapy for conditions such as diabetes, cancer, cardiovascular disease, and neurological disorders [4].

Over the last several decades, significant advancements have been made in the field of nanomaterials and surface modification. These advancements have been detected and achieved in various fields such as biomedicine, biotechnology, catalysis, and magnetic chemistry [6, 7]. MNPs such as nickel, cobalt, and iron have garnered significant attention [8–11]. MNPs are frequently coated with molecules or polymers to improve their stability in aqueous and physiological environments. Untreated MNPs tend to aggregate when they interact with biological molecules. Utilizing the coating method can boost the colloidal stability of magnetic nanoparticles (MNPs), allowing them to persist and be difficult to remove during blood circulation for an extended duration [12]. The prolonged circulation time of magnetic nanoparticles (MNPs) within the bloodstream allows for extended interaction with biological components, potentially increasing opportunities for targeted binding or modifications. This increased exposure and interaction within the biological environment enhance the likelihood of MNPs undergoing functionalization processes for specific biomedical applications [5, 12]. Iron oxide (Fe_3_O_4_) MNPs, for example, are frequently prepared in different morphologies, such as spherical or hollow spherical structures of less than 100 nm in size, which enable drug delivery in the human body [13–15].

Graphene oxide (GO) has attracted attention in several research fields, such as solar cells, bio-sensing, membrane technology, and energy storage, due to its excellent chemical, physical, and mechanical characteristics [16–18]. Also, GO possesses a characteristic property such as amphiphilic, which enables it to penetrate cell membranes [19]. Furthermore, the non-covalent GO bonding facilitates the functionalization of its surface sheets and enhances the conjugation process due to π–π interaction [16].

GO has been synthesized via different processes such as chemical vapor deposition, thermal and mechanical exfoliation, radiation-based, and pyrolysis [20–22]. The modified Hummer method is widely acknowledged as one of the methods that have been used to synthesize GO, yielding multiple oxygen-containing groups [16, 23].

Due to the non-covalent bonding of GO, and functional groups including carboxylic, epoxide, and hydroxyl, the functionalization of GO with Fe_3_O_4_ is beneficial [24–28]. Furthermore, MNPs such as Fe_3_O_4_ coated with polymers such as chitosan and dextran enhance the aggregation and significantly decrease the composite surface energy [29]. GO/Fe_3_O_4_, as an example, was loaded with chitosan to prepare GO/Fe_3_O_4_/Chitosan nanocomposite by linking the amino groups of Fe_3_O_4_/Chitosan with the GO carboxylic group [30, 31]. GO/Fe_3_O_4_/Chitosan nanocomposite demonstrates a significant degree of bio-activity as an excellent platform for different drug delivery applications [32, 33].

Digitonin (DIG) shows a significant potential to be used in drug delivery applications [34, 35]. Therefore, A DIG-based GO/Fe_3_O_4_ nanocomposite may result in a reduction of the interlayer spacing between the MNPs and GO, which may enhance the nanocomposite effective surface area [36–42].

In this study, the GO nanosheets and the Fe_3_O_4_ were prepared using the modified Hummer and co-precipitation methods, respectively. DIG/GO and a dual-targeted platform of DIG/GO/Fe_3_O_4_ nanocomposite was prepared. The physiochemical properties of the synthesized samples were investigated, including the surface morphology, and structural and chemical characteristics. Furthermore, the antioxidant activity of the prepared samples was measured.

## Experiment

### Materials

Digitonin (DIG), sodium hydroxide (NaOH), sodium nitrate (NaNO_3_), hydrochloric acid (HCl), pure graphite, ferric chloride hexahydrate (FeCl_3_.6H_2_O), potassium permanganate (KMnO_4_), sodium tripolyphosphate (STP, Na5P3O10), ferrous chloride tetrahydrate (FeCl_2_.4H_2_O), hydrogen peroxide (H_2_O_2_), ethylene, DMF, and DMSO are obtained from Sigma-Aldrich, USA.

### Preparation of Fe_3_O_4_ MNPs

The co-precipitation method was carried out to prepare the Fe_3_O_4_ MNPs. Initially, 2.59 g of FeCl_3_.6H_2_O and 1.59 g FeCl_2_.6H_2_O were dissolved individually in 100 ml HCl (37%) and mixed under mechanical stirring conditions at room temperature for about 15 min, the temperature was increased to 40 °C. Consequently, 20 ml of 3 M NaOH was gradually added dropwise for 1 h under continuous stirring. Finally, the resulting solution underwent centrifugation, leading to the magnetic collection of the precipitate. The precipitate was then subjected to washing with deionized water and ethanol, followed by drying in an oven set at a temperature of 65 °C for 24 h.

### Preparation of GO nanosheets

The following steps are involved in producing GO nanosheets using the modified Hummer's method [43]. In the first step, 2.5 g NaNO_3_ was dissolved in 112 ml H_2_SO_4_ (96%) under mechanical stirring for 20 min. Subsequently, 4.6 g graphite powder was gradually added to the solution and continuously stirred for 10 min. In the second step, 15 g of KMnO_4_ was added to the mixture solution under vigorous stirring at a temperature of 0 °C for 1 h. Notably, the temperature was gradually increased to 40 °C while the mixture was then stirred for 2 h. In the third step, a solution of 20 ml of H_2_O_2_ (30%) and 240 ml DI water was slowly added to the final solution to remove the excess KMnO_4_. In the fourth step, a solution of yellow paste was produced and subsequently subjected to centrifugation at 6000 rpm for 10 min. The resulting paste was subjected to multiple washing processes using diluted HCl (10%) and DI water. Finally, after the completion of the washing process, a dark brown graphite oxide paste was successfully obtained. This paste was then subjected to a drying process in an oven with a temperature of 70 °C for 24 h. GO nanosheets were acquired in the form of a powdery substance.

### Preparation of DIG/GO nanocomposite

The first solution was prepared by dissolving 200 mg of DIG in 0.10 ml AcOH and 5 ml of DI water. The second solution of GO was obtained by dissolving 25 mg GO in 10 ml DI water and sonicated for 40 min. The DIG solution was added to the GO solution under the influence of ultrasonic irradiation for 15 min. Finally, the DIG/GO nanocomposite solution was synthesized, centrifuged at 600 rpm for 15 min, and dried in the oven at 60 °C for a period of 2 h.

### Preparation of DIG/GO/Fe_3_O_4_ nanocomposite

The DIG/GO/Fe_3_O_4_ nanocomposite preparation was carried out as follows:

First step, a mixture of 25 mg of Fe_3_O_4_ MNPs and 25 mg of GO nanosheets were added to 10 ml DI water under the influence of sonication probe for 30 min in order to prepare a homogenous solution of GO/Fe_3_O_4_ nanocomposite. Second step, 200 mg of DIG was dissolved in a solution composed of 0.10 ml AcOH and 5 ml of DI water to prepare a solution of DIG. In the third step, the GO/Fe_3_O_4_ nanocomposite solution was added to the DIG solution using the sonication probe for 20 min. The fourth step involved the preparation of a cross-linking solution. To achieve this, 1 g of Na_5_P_3_O_10_ was mixed in 100 ml of DI water. Subsequently, 83.3 ml of this solution was added to 16.7 ml of ethanol and the mixture was subjected to mechanical stirring for 1 h. In the fifth step, the solution obtained from the third step was added to the cross-linking solution from the fourth step under mechanical stirring for 1 h. Finally, the resultant solution, namely the DIG/GO/Fe_3_O_4_ nanocomposite, underwent centrifugation at 6000 rpm for 15 min and dried in an oven set at 60 °C for 2 h.

### Characterization techniques

The structural and surface morphology properties of the prepared samples were analyzed using a Powder X-ray Diffractometer (XRD, Malvern Panalytical Ltd) and Scanning Electron Microscopy (SEM, Quanta FEG 450), respectively. The Fourier transform infrared spectroscopy was performed on the samples using the Thermo Scientific Nicolet iS10 FT-IR Spectrometer in the region of (250–4000) cm^−1^. Optical absorbance measurements were executed using Agilent Cary 5000 UV–Vis–NIR spectrophotometer.

## Results and discussion

### Surface morphology

**Fig. 1:**
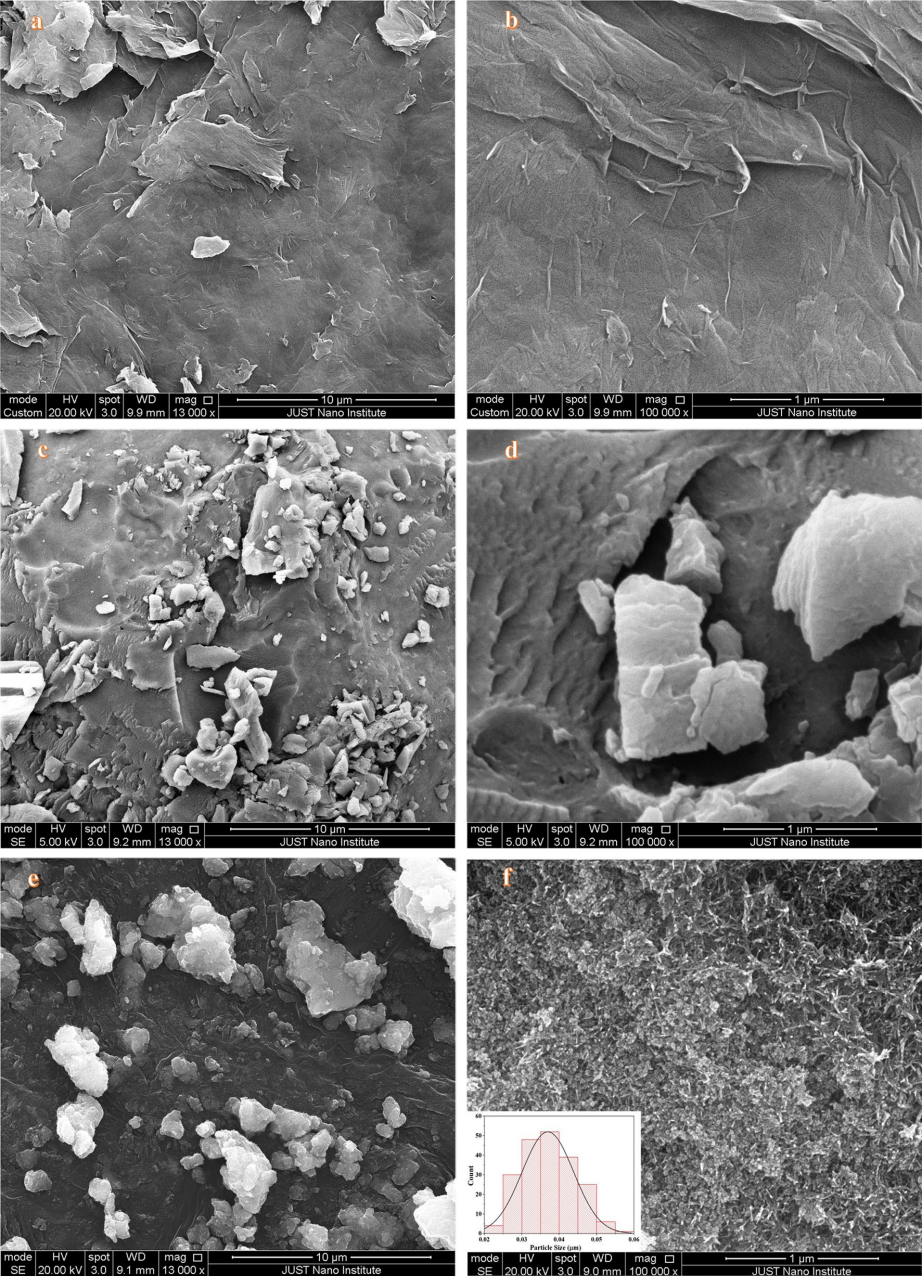
The SEM micrographs of the prepared samples. **a**, **b** GO, **c**, **d** DIG, and **e**, **f** Fe_3_O_4_

**Fig. 2:**
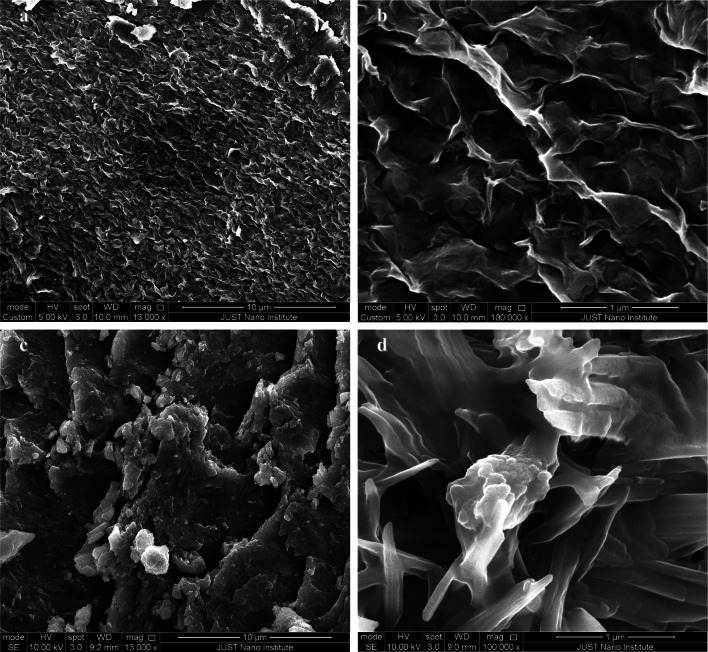
The SEM micrographs of the as-prepared nanocomposite samples. **a**, **b** DIG/GO nanocomposite, **c**, **d** DIG/GO/Fe_3_O_4_ nanocomposite

The SEM images presented in Fig. 1 illustrate the morphology of the synthesized samples. Figure 1a, b provides insight into the formation of multilayer structures of GO nanosheets. Additionally, it is observed that flakes of different sizes formed after the drying process of GO nanosheets, particularly at the edges of the sheet structure. The presence of small-scale GO nanosheets and multiple sheets facilitates the dispersion of Fe_3_O_4_ MNPs within the stacked layers, thereby enhancing their potential for drug delivery. Figure 1c, d depicts the nanoscale aggregation of Fe_3_O_4_ MNPs, showcasing a range of various sizes. The average size of the Fe_3_O_4_ MNPs was calculated as 38.64 nm using the Gaussian distribution of the MNPs given inset in Fig. 1d using the SEM image. Figure 1e, f illustrates the surface morphology of DIG, indicating a low porosity and the existence of multiple stacked layers, as well as the presence of observed aggregates on the surface. Additionally, the SEM images show the formation of clusters of DIG molecules with varying sizes. Figure 2 illustrates the surface morphologies of the composite material DIG/GO before and after incorporating Fe_3_O_4_ MNPs. A uniform array of GO multilayers and DIG composite can be observed in Fig. 2a, b. A more detailed investigation reveals a substantial level of roughness detected on the surface, which suggests that DIG material is intercalated between the GO nanosheet layers. The structure serves as a platform for the dispersed and impeded Fe_3_O_4_ nanoparticles.

Figure 2c, d shows the presence of non-uniform composites resulting from loading Fe_3_O_4_ MNPs onto the DIG/GO nanocomposite. In addition, it is quite clear that grains of varying sizes are arranged in a stacked formation, resulting in a multi-layered array. At a smaller scale, the loading of Fe_3_O_4_ MNPs onto GO layers that are intercalated into the DIG material can be observed. The DIG material acts as a platform for intercalating GO sheets and incorporating Fe_3_O_4_ nanoparticles, which have a high molecular weight and large mass ratio, into preparing DIG/GO/Fe_3_O_4_ nanocomposite.

### Structural analysis

**Fig. 3:**
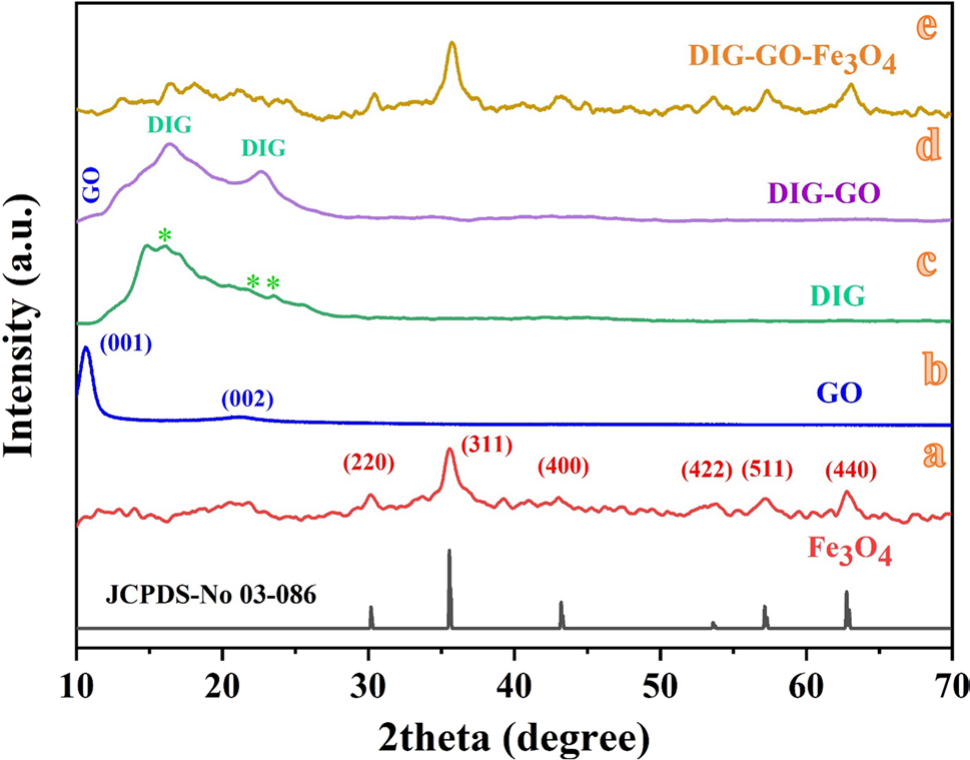
The X-ray diffraction pattern of **a** GO, **b** Fe_3_O_4_ nanoparticles, **c** DIG, **d** DIG/GO nanocomposite, and **e** DIG/GO/Fe_3_O_4_ nanocomposite

The crystal structures of the samples were examined utilizing the Powder X-ray data analysis system, as illustrated in Fig. 3. Figure 3a demonstrates the GO nanosheet diffraction patterns. Sharp and weak peaks at 2θ = 10.5° and 21.3°, respectively, can be observed, suggesting the presence of crystallographic planes characterized by Miller indices (hkl) of (001) and (002) [23]. The sharp peak observed in the spectrum can be ascribed as the distinctive peak of the GO nanosheets, whereas the relatively weak peak, which is approximately 1/8th of the intensity of the characteristic GO peak, can be linked to a GO nanosheets [44]. The XRD patterns of Fe_3_O_4_ MNPs is shown in Fig. 3b. All of the diffraction patterns observed at 2θ = 30.3°, 35.6°, 43.3°, 53.6°, 57.3°, and 63.0°, which correspond to the Miller indices (220), (311), (400), (422), (511), and (440) respectively, are consistent with the diffraction patterns reported for Fe_3_O_4_ MNPs (JCDP card No.03-086) [45]. No impurity and secondary peaks were detected in the XRD analysis, indicating that the Fe_3_O_4_ MNPs in the system are free from contamination and confirming the successful formation of magnetite [45–47]. The XRD pattern of the DIG-GO nanocomposite, as presented in Fig. 3d, exhibits the major diffraction pattern of GO at an angle of 10.5°. Additionally, there are notable peaks observed at angles of 22.9°, 16.2°, and 14.9°, denoted by (∗), which indicate the presence of DIG with a higher intensity in comparison to the GO nanosheets. This result indicates that the DIG remained unchanged while forming the DIG/GO nanocomposite. Additionally, the GO nanosheets dispersed on DIG, resulting in a functionalization process between GO nanosheets and DIG. This process leads to the formation of the DIG/GO nanocomposite. Interestingly, the XRD patterns of the nanocomposite system DIG/GO/Fe_3_O_4_, as shown in Fig. 3e, demonstrate minimal alterations compared to the XRD pattern of Fe_3_O_4_ MNPs. This observation suggests that the Fe_3_O_4_ MNPs crystallinity structure remains unchanged after the composition process. Also, the dispersion of Fe_3_O_4_ MNPs on the DIG-GO is observed. Furthermore, Fig. 3e displayed a weak peak associated with GO structure, which is attributed to the low ratio of GO in the mixture. This observation also suggests to well dispersion of GO in the composite.

### Physical analysis

**Fig. 4:**
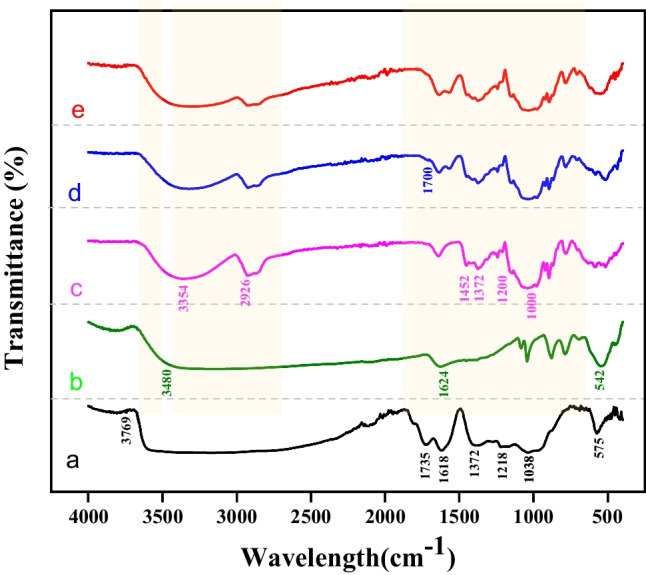
The FTIR of **a** GO, **b** Fe_3_O_4_ nanoparticles, **c** DIG, **d** DIG-GO nanocomposite, and **e** DIG/GO/Fe_3_O_4_ nanocomposite

Figure 4 depicts the FTIR for GO, ${{\text{Fe}}}_{3}{{\text{O}}}_{4}$, DIG, and ${\text{DIG}}/{\text{GO}}/{{\text{Fe}}}_{3}{{\text{O}}}_{4}$ samples. Figure 4a illustrates the FTIR transmission of the GO major bands between 3769 and 3000 cm^−1^ which corresponds to the hydroxyl groups and O–H stretching [48]. The peak located at 1735 cm^−1^ is assigned to the carbonyl and carboxylic groups presented in GO. Also, three peaks observed between 1038 and 1218 (cm^−1^) were associated with the alkoxy and epoxy groups [46, 49]. Figure 4b shows the FT-IR spectrum of the Fe_3_O_4_ MNPs. The main peak was observed at 542 cm^−1^, which is attributed to the stretching vibration mode attributed to the metal–oxygen Fe–O bonds in the crystalline lattice of ${{\text{Fe}}}_{3}{{\text{O}}}_{4}$ [50, 51]. Moreover, the presence of hydroxyl groups is linked to a wide band at $3480\,{{\text{cm}}}^{-1}$ and band at $1624\,{{\text{cm}}}^{-1}$, were both associated to OH-stretching and OH-bending, respectively [52]. The FT-IR spectrum of ${\text{DIG}}$ as in Fig. 4c illustrates a broad band at $3410\,{{\text{cm}}}^{-1}$ corresponding to –OH stretching vibration, and a band at $2926\,{{\text{cm}}}^{-1}$ associated to C–H stretching and bending vibration [53, 54]. Also, two absorption peaks were observed at $1452\, {{\text{cm}}}^{-1}$, and $1372 \,{{\text{cm}}}^{-1}$ associated to C–H deforming vibration. The absorption peaks observed in the FT-IR profile of the ${\text{DIG}}$ in the spectral range of 1000–1200 cm^−1^ are associated to C–O–H stretching vibration, and C–O–C glycosidic band vibration [55]. The FT-IR spectrum of the as-prepared ${\text{DIG}}/{\text{GO}}$ nanocomposite as shown in Fig. 4d reveals major peak at $3769\,{{\text{cm}}}^{-1}$ and $575\,{{\text{cm}}}^{-1}$ demonstrating the presence of the GO nanosheets on the DIG surface [46]. The C=O stretching of the tertiary amide may be accountable for a new peak at $1700\,{{\text{cm}}}^{-1}$, which demonstrates the successful functionalization of DIG with GO [46]. The ${\text{DIG}}/{\text{GO}}/{{\text{Fe}}}_{3}{{\text{O}}}_{4}$ spectrum as in Fig. 4e demonstrates the ${{\text{Fe}}}_{3}{{\text{O}}}_{4}$ MNPs have been effectively loaded on the GO/DIG nanocomposite [46].

### Optical absorbance

**Fig. 5:**
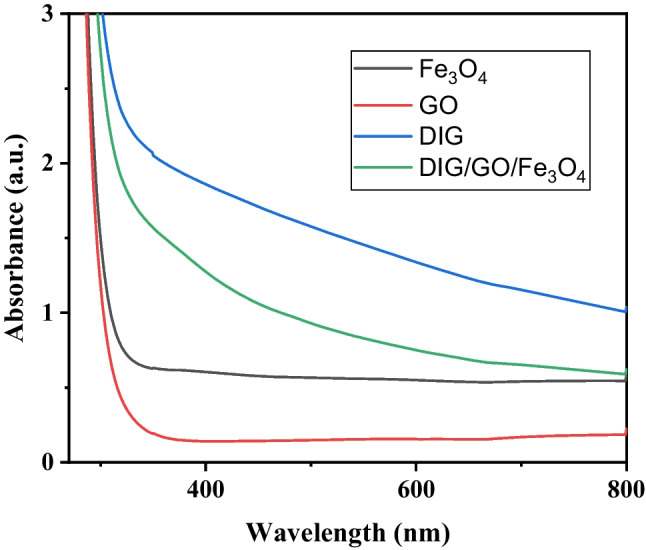
The absorbance spectra for Fe_3_O_4_ MNPs, GO, DIG, and the DIG/GO/Fe_3_O_4_ composite

The investigation of the absorbance of the spectra of ${{\text{Fe}}}_{3}{{\text{O}}}_{4}$ MNPs, ${\text{GO}}$, DIG, and ${\text{DIG}}/{\text{GO}}/{{\text{Fe}}}_{3}{{\text{O}}}_{4}$ composite with distinctive patterns were presented in Fig. 5. The spectra emerged, shedding light on the optical properties of these materials. Notably, the absorbance spectrum of ${{\text{Fe}}}_{3}{{\text{O}}}_{4}$ MNPs illustrated a gradually increasing in the absorbance starting around 700 nm, with a sudden surge beyond 355 nm, demonstrating its distinct absorption behavior. Conversely, the absorbance spectrum of ${\text{GO}}$ showed a sharp onset at approximately 400 nm, indicative of its unique electronic transitions. DIG, On the other hand, exhibited a continuous rise in absorbance from 800 to 350 nm, culminating in a sudden increase. The absorbance spectrum of the ${\text{DIG}}/{\text{GO}}/{{\text{Fe}}}_{3}{{\text{O}}}_{4}$ composite revealed a nuanced interplay of the individual components with a gradual rise from 800 nm and a more increase from 700 nm, suggesting the influence of ${{\text{Fe}}}_{3}{{\text{O}}}_{4}$ MNPs and ${\text{GO}}$ in the visible region, while the composite exhibited absorbance characteristics positioned between the individual components.

### Antioxidant activity

The determination of the antioxidant activity of the compounds is conducted using the 2-diphenyl-1-picrylhydrazyl (DPPH) and 2,2-azino-bis-3-ethylbenzthiazoline-6-sulfonic acid (ABTS)radical scavenging assay methods, as outlined in the literature [56–58]. The scavenging activity was computed by the equation given as [59]:

$$\%\, \text{Scavenging activity }=\frac{({A}_{S}-{A}_{C})}{{A}_{C}}*100$$

The absorbance of the control is denoted as A_c_, while the absorbance of the extract is defined as A_s_. The IC_50_ values were calculated by evaluating the relationship between the percentage of antiradical activity and the concentration of the compounds.

#### DPPH method

The DPPH of the extracts was carried out and compared to α-tocopherol and ascorbic acid in order to determine their total capacity [60]. In this manner, an amount of 2.0 ml of 0.1 mM of DPPH solution was added to 2.0 ml of the extract at various concentrations between 3.12 and 15.6 ($\times$10^–3^ mg/ml). The resultant solution was incubated in dark conditions for 30 min, and the absorbance was determined against methanol as a blank at wavelength of 517 nm.

#### ABTS method

**Fig. 6:**
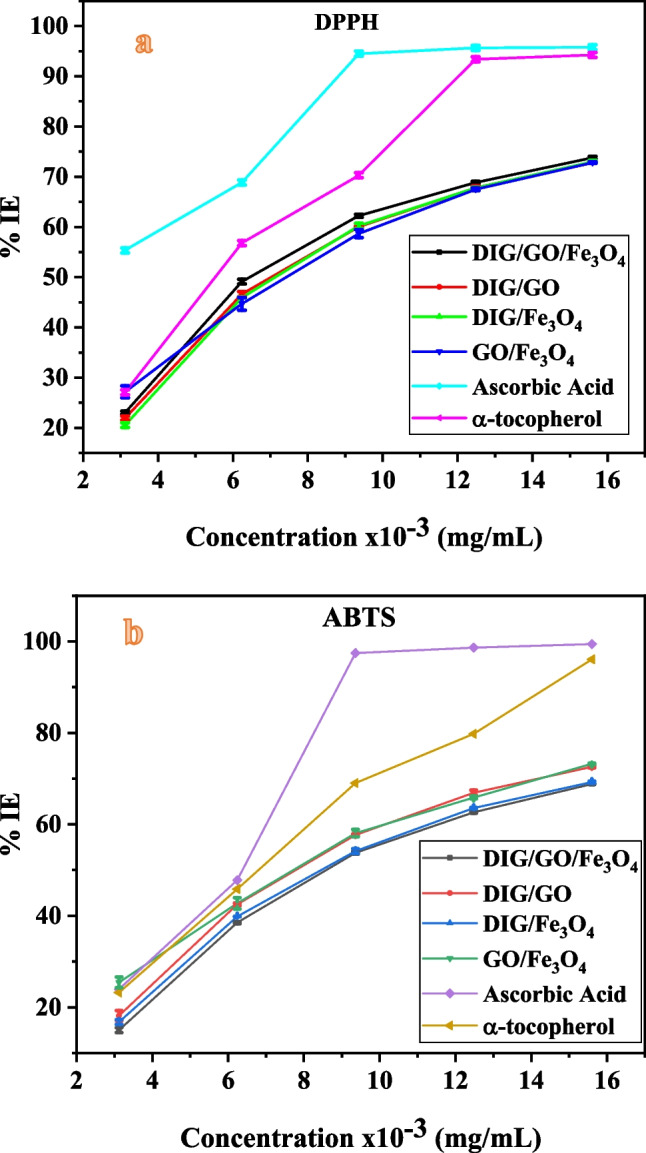
Antioxidant activities of the compounds and positive controls (α-tocopherol and ascorbic acid) by using **a** DPPH, and **b** ABTS assays

The determination of antioxidant measurements was conducted according to the power of the ABTS^**·+**^ method [56–58]. The cation radical ABTS^**·+**^ was added to distilled water. After this, 3 ml of the resultant ABTS solution was mixed with 1 ml extract with different amounts between 3.12 and 15.6 ($\times$ 10^–3^ mg/ml). The absorbance was measured at the wavelength of 734 nm using a UV–vis spectroscopic. The absorbance was carried out every 5 min. The ABTS capacity was compered to α-tocopherol and ascorbic acid standards. The concentration of the compound that causes a 50% reduction of the DPPH and ABTS (IC_50_) was measured to interpret the findings from both DPPH and ABTS methods. Using the linear regression, the IC_50_ was evaluated by plotting the percentage of the antiradical activity versus the concentration level of compounds as shown in Fig. 6.

In the present study, the assessment of the compounds' ability to scavenge free radicals was conducted using the ABTS and DPPH free radical scavenging methods. Screening results revealed that the antioxidant activities of all compounds exhibited a concentration-dependent pattern as in Fig. 6. The IC_50_ values for each compound are listed in Table 1. In comparison to the positive controls used in the present study, the four compounds exhibited significant antioxidant activity on both assay methods. Among the prepared samples, DIG/GO/Fe_3_O_4_ nanocomposite demonstrates a significant high antioxidant activity in both DPPH^**·**^ and ABTS^**·+**^ tests. Furthermore, the results show that the DIG, GO, and Fe_3_O_4_ MNPs synergistically interact in terms of their ability to free radicals scavenging. This phenomenon can be attributed to the tendency of spherical Fe_3_O_4_ MNPs and lamellar GO to aggregate into significant clusters in aqueous solutions, thereby impeding their capability to effectively scavenge radicals [61]. When both DIG/GO/Fe_3_O_4_ nanocomposite were instantaneously exist in an aqueous solution, the spherical Fe_3_O_4_ MNPs might be successfully loaded onto the GO lamellae and intercalated to reduce agglomeration. This resulted in an increased number of active sites presented for interaction with free radicals, thereby enhancing the capacity for scavenging free radicals.

**Table 1:** IC_50_ values of the compounds and positive controls using DPPH and ABTS methods

Sample	IC_50_ (DPPH) (mg/mL) × 10^–3^	IC_50_ (ABTS) (mg/mL) × 10^–3^
DIG/GO/Fe_3_O_4_	6.86 ± 0.04	5.69 ± 0.02
DIG/GO	7.21 ± 0.03	7.76 ± 0.09
DIG/Fe_3_O_4_	7.31 ± 0.02	8.45 ± 0.04
GO/Fe_3_O_4_	7.03 ± 0.11	7.34 ± 0.05
Ascorbic acid	1.80 ± 0.06	1.90 ± 0.06
α-tocopherol	2.30 ± 0.04	1.80 ± 0.01

## Conclusion

In the present study, the Fe_3_O_4_ MNPs and GO nanosheets were combined to form the GO/Fe_3_O_4_ nanocomposite. The GO/Fe_3_O_4_ nanocomposite was then consequently conjugated with DIG to prepare a nanocomposite platform system, known as DIG/GO/Fe_3_O_4_ nanocomposite. SEM images illustrate the formation of multilayer GO nanosheets, the dispersion of Fe_3_O_4_ MNPs within the GO layers, and the surface characteristics of the DIG. SEM images also showed the existence of a porous structure in the DIG/GO/Fe_3_O_4_ nanocomposite. In addition, the aggregation of the GO/Fe_3_O_4_ MNPs coated with DIG resulted in the formation of the DIG/GO/Fe_3_O_4_ nanocomposite. The validation of the structural analysis of the prepared samples was performed via XRD analysis. The main diffracted peaks for Fe_3_O_4_ MNPs are depicted. Moreover, the major characteristic peaks of GO nanosheets were observed and associated to the (001) and (002) crystallographic planes. The presence of crosslinking between the GO nanosheet layers and the Fe_3_O_4_ MNPs in the GO/DIG/Fe_3_O_4_ nanocomposite is demonstrated by the examined FT-IR vibrational modes. The antioxidant activity of the prepared samples was measured, and it was found that the DIG/GO/Fe_3_O_4_ nanocomposite exhibited significantly high antioxidant activity in both the DPPH^**·**^ and ABTS^**·+**^ tests. Thus, the obtained results indicate that this novel composite has prospective uses in biomedical formulations due to its promising antioxidant activity, which may help address oxidative stress-related diseases. Additionally, its structural integrity suggests applicability in catalysis, sensing technologies, and environmental remediation.

## Data Availability

The data that support the findings of this study are available from the corresponding author, B.A., upon reasonable request.
